# Silencing of METTL16 protects granulosa cells from the cisplatin-induced ferroptosis in premature ovarian failure

**DOI:** 10.1038/s41420-026-03081-3

**Published:** 2026-04-19

**Authors:** Jing Xiong, Ling He, Yongjing Zhang, Lifang Li

**Affiliations:** Department of Obstetrics and Gynecology, the Second Xiangya Hospital of Central South University, Changsha, Hunan China

**Keywords:** Apoptosis, Gene silencing

## Abstract

Premature ovarian failure (POF) and insufficiency induced by cisplatin are common complications associated with gynecological diseases. This study aims to investigate the role of N^6^-methyladenosine (m^6^A) methyltransferase METTL16 in cisplatin-induced ovarian granulosa cells ferroptosis. In cisplatin-treated ovarian tissue, the level of METTL16 was significantly elevated. Furthermore, METTL16 was also upregulated in cisplatin-stimulated granulosa cells. Functionally, silencing METTL16 inhibited iron accumulation and lipid peroxidation, while alleviating mitochondrial injury. Mechanistically, METTL16 was found to target NRF2, negatively regulating its RNA stability, and YTHDF2 facilitated the degradation of NRF2 mRNA. In summary, the METTL16/YTHDF2/NRF2 axis regulates ferroptosis in cisplatin-stimulated granulosa cells in POF. This study suggests that METTL16 may serve as a promising immunotherapeutic target for POF.

## Introduction

Premature ovarian failure (POF) affects a woman’s fertility, leading to irregular menstruation and low estrogen levels [1]. Epidemiological studies have shown that the prevalence of POF is about 10% with an upward trend in some countries and regions. The pathologic basis of POF is complex and varied, including genetic factors, chromosomal abnormalities, immunological factors, enzyme defects, and gonadotropin dysfunction, as well as radiotherapy and chemotherapy [2]. Cisplatin (also known as Cis-dichlorodiammineplatinum(II), CDDP) acts as a widely used chemotherapy drug with cytotoxic effects, which directly damage germ cells, including oocytes and granulosa cells in the ovaries [3]. This damage leads to accelerated follicle injury and follicles decreasing, which triggers POF.

N^6^-methyladenosine (m^6^A) is an RNA methylation modification, which affect processes such as mRNA stability, splicing and degradation, thereby regulating gene expression [4, 5]. Abnormal regulation of m^6^A modification impacts cellular functions through various mechanisms. m^6^A modification plays critical roles in oocyte maturation, ovarian development, and embryo development. Besides, m^6^A modification is intricately involved in the pathogenesis of premature ovarian failure (POF), polycystic ovary syndrome (PCOS), and endometriosis [6].

Ferroptosis is an iron-dependent form of cell death that is involved in a variety of cellular biological processes, such as redox balance, iron metabolism, mitochondrial activity, and disease-related signaling pathways [7, 8]. The main feature of ferroptosis is the intracellular accumulation of lipid peroxidation products and lethal reactive oxygen species [9]. Abnormal iron metabolism and lipid metabolism damage ovarian granulosa cells and follicles by inducing ferroptosis, thus impairs ovarian function [10]. J.Q. Chen (2024) reported that cisplatin could activate genes associated with ferroptosis, and human umbilical cord mesenchymal stem cells (hUCMSCs) alleviates cisplatin-induced POF by suppressing the ferroptosis and reducing fibrosis-related factors (α-SMA, COL-I) [11]. L Zhang (2024) reported that ferrostatin-1 (Fer-1), a selective ferroptosis inhibitor, could decrease the extent of ferroptosis and ovarian toxicity induced by Cis-dichlorodiammineplatinum(II), including iron accumulation, excessive ROS accumulation and mitochondrial dysfunction [12]. It can be seen from the present reports that ferroptosis is deeply involved in the occurrence of POF.

The present study aims to analyze and investigate the role of METTL16 on cisplatin-induced ovarian injury and ferroptosis. Through partial in vivo and in vitro experiments, the research elucidated the regulatory mechanism of METTL16 on cisplatin-induced ovarian injury and ferroptosis. The findings might offer a novel protective strategy for POF treatment.

## Results

### METTL16 expression was triggered by the cisplatin-stimulated POF

**Fig. 1:**
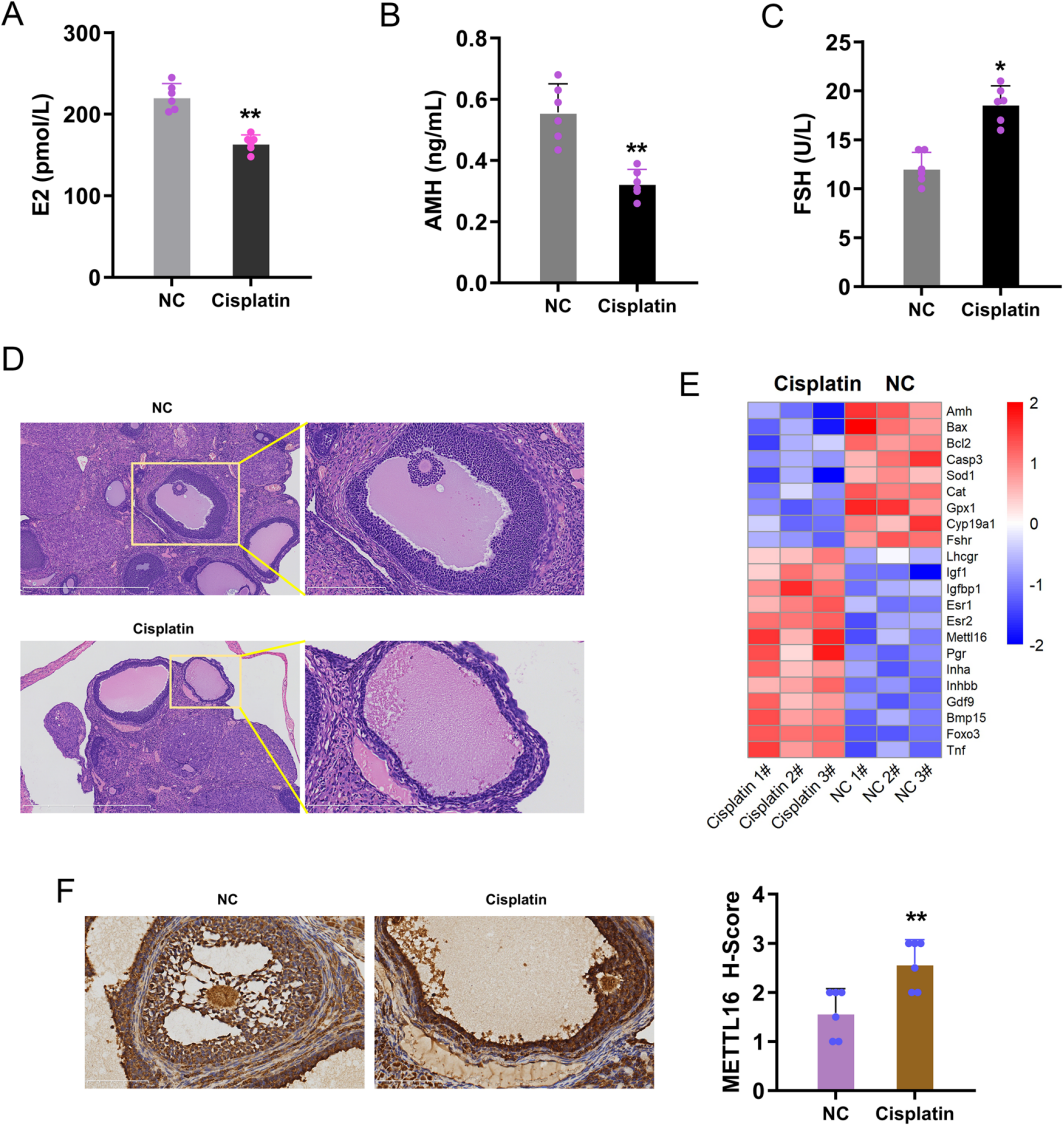
METTL16 expression was triggered by the cisplatin-stimulated POF. **A** The estradiol, **B** anti-miller tube hormone (AMH), and **C** FSH level were tested by ELISA. **D** Hematoxylin-Eosin (HE) staining in the cisplatin-induced POF mice ovarian tissue. **E** Heatmap showed the differentially expressed genes/RNAs in cisplatin-induced POF animal model ovarian tissue. **F** Immumohistochemical (IHC) staining of METTL16 in POF animal model ovarian tissue. Data are presented as mean ± SD (*n* = 6 independent biological replicates per group). *p < 0.05; **p < 0.01.

In cisplatin-stimulated POF animal model tissues, estradiol levels (Fig. 1A) and anti-Müllerian hormone (AMH) (Fig. 1B) were reduced in the ovarian tissues. Besides, the FSH (Fig. 1C) level was increased in cisplatin-stimulated animal model ovarian tissue. Ovarian tissue was obtained from cisplatin-induced premature ovarian failure (cisplatin-POF) mice (Fig. 1D). In the sequencing data, the highly or lowly expressed genes/RNAs were tested in the tissue samples with cisplatin (Fig. 1E). In the highly expressed genes, the METTL16 was tested in the tissue samples (Fig. 1F). Overall, the findings revealed that METTL16 expression was triggered by the cisplatin-stimulated POF.

### METTL16 was up-regulated in cisplatin-stimulated granulosa cells

**Fig. 2:**
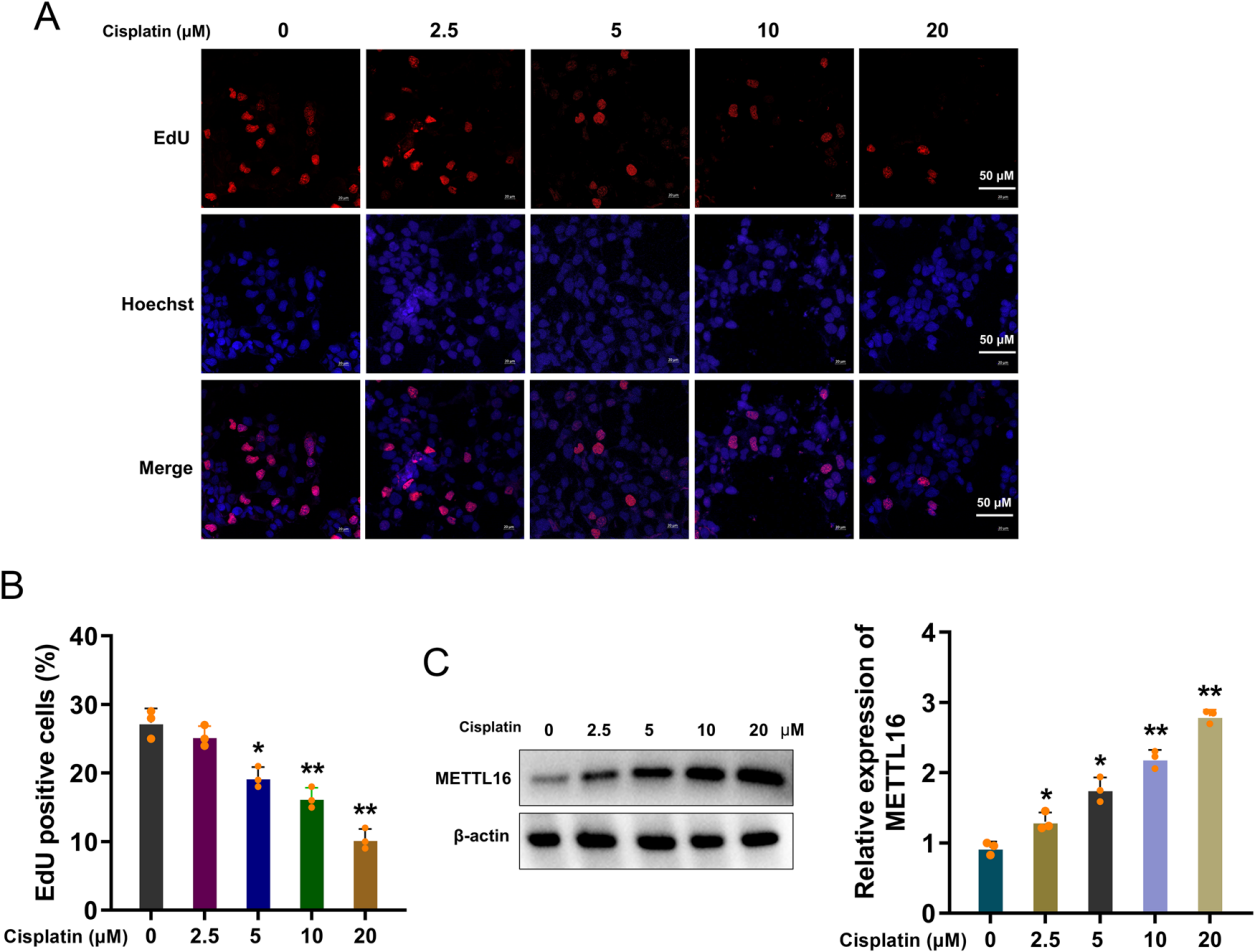
METTL16 was up-regulated in cisplatin-stimulated granulosa cells. **A** Proliferation assay by EdU was performed in the cisplatin-stimulated granulosa cells. **B** The quantitative analysis of EdU positive cells. **C** The METTL16 protein level was tested in cisplatin-stimulated granulosa cells. The quantitative analysis of METTL16 protein. Data are presented as mean ± SD (*n* = 3 independent biological replicates per group). *p < 0.05; **p < 0.01.

To explore the role and function of cisplatin on granulosa cells, the proliferation assay was performed. In the cisplatin-stimulated granulosa cells, the proliferative characteristic was reduced upon cisplatin treatment (Figs. 2A, B). Given that METTL16 was up-regulated in cisplatin-stimulated POF animal model ovarian tissue, the level of METTL16 in cisplatin-stimulated granulosa cells was tested. Western blot showed that METTL16 protein level was up-regulated in cisplatin-stimulated granulosa cells (Fig. 2C). Therefore, the data indicated that METTL16 was up-regulated in cisplatin-stimulated granulosa cells, which was closely correlated to the cisplatin stimulation.

### METTL16 silencing repressed the iron accumulation

**Fig. 3:**
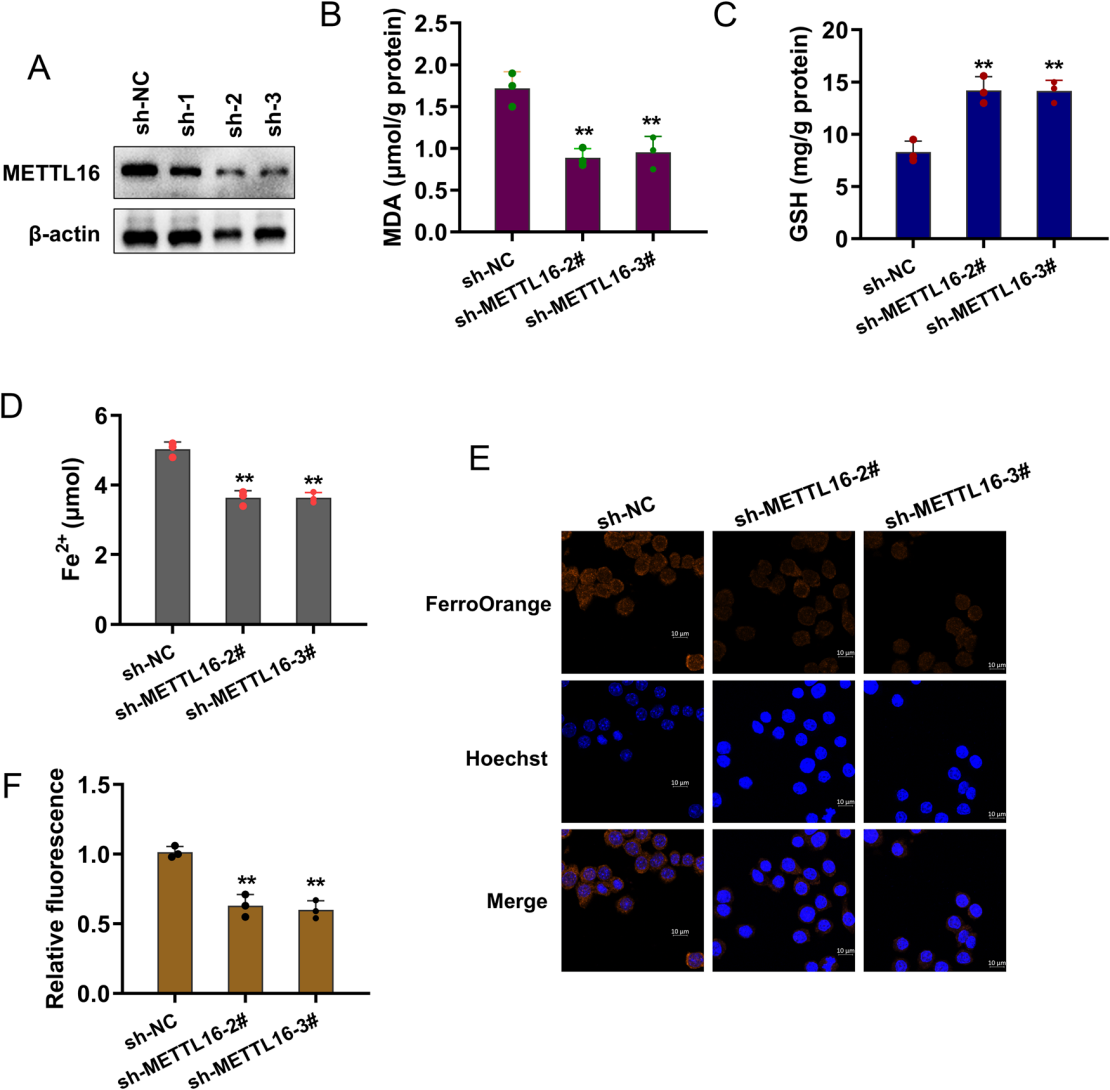
METTL16 silencing repressed the iron accumulation. **A** The METTL16 silencing was performed using the short hairpin oligonucleotides (sh-NC, sh-METTL16-1, sh-METTL16-2, sh-METTL16-3). **B** The MDA level was tested by the MDA kit. **C** The GSH level was tested by the GSH kits. **D** Fe^2+^ concentration was tested by the iron analysis kit. **E**, **F** The level of labile ferrous ion in cisplatin-stimulated granulosa cells was tested by FerroOrange fluorescence prob with METTL16 silencing. Data are presented as mean ± SD (*n* = 3 independent biological replicates per group). *p < 0.05; **p < 0.01.

In the cellular assay, the role of METTL16 was tested in the cisplatin-stimulated granulosa cells. The METTL16 silencing was performed using the short hairpin oligonucleotides (Fig. 3A). To validate the role of METTL16 in ferroptosis of granule cells, the relevant assays were performed as following. To assess the characteristics of iron deposition, the results showed that METTL16 silencing reduced the MDA (Fig. 3B), promoted GSH levels (Fig. 3C), and inhibited the Fe^2+^ concentration (Fig. 3D). Besides, to investigate the cellular iron accumulation, the FerroOrange fluorescent probe assay indicated that METTL16 silencing reduced the accumulation of labile iron ions in the cytoplasm (Figs. 3E, F). Results indicated that METTL16 silencing repressed the iron accumulation.

### METTL16 silencing repressed the lipid peroxidation and alleviated the mitochondria injury

**Fig. 4:**
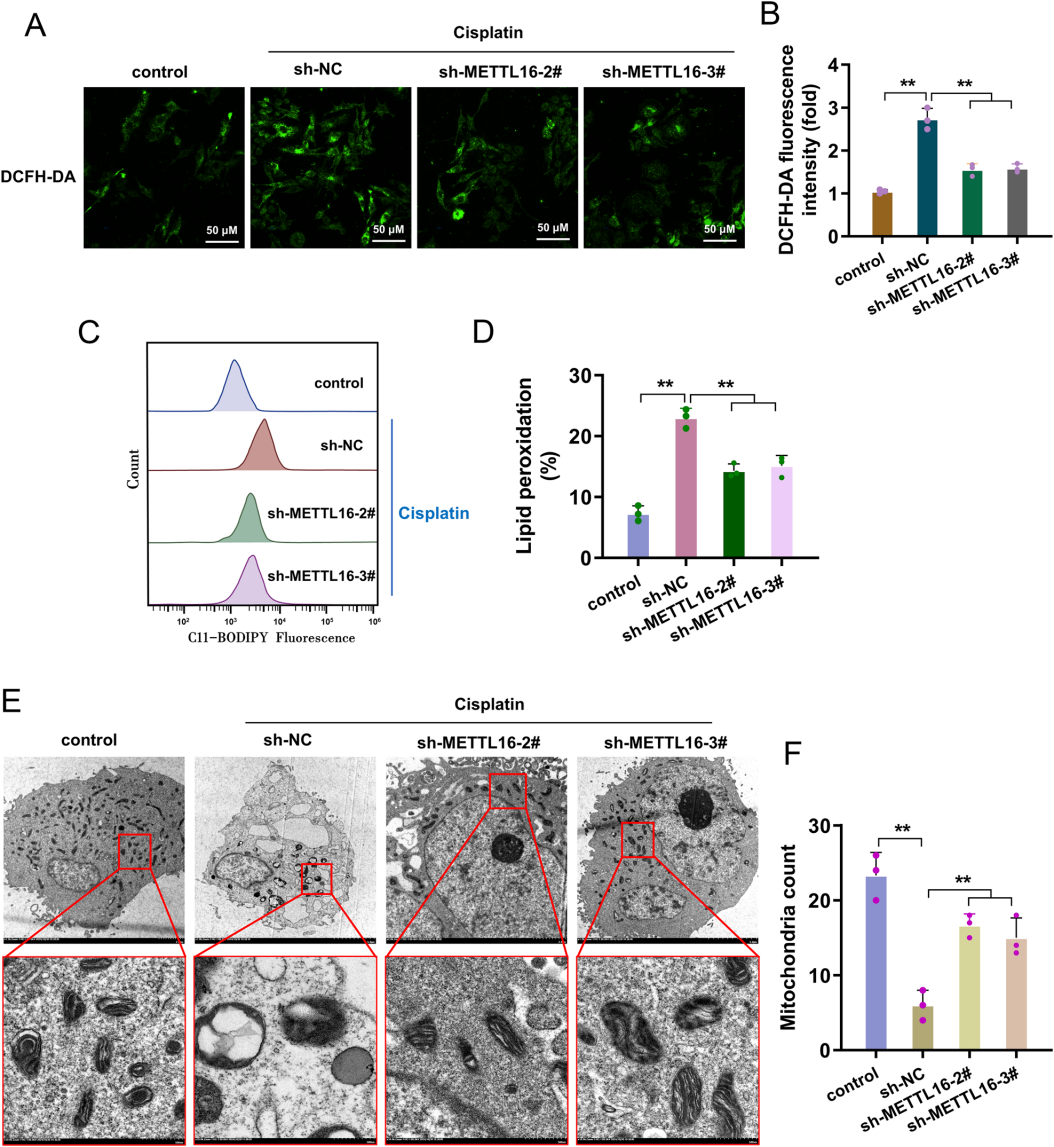
METTL16 silencing repressed the lipid peroxidation and alleviated the mitochondria injury. **A** Representative DCFH-DA fluorescence images for intracellular ROS in granulosa cells under cisplatin stimulation (10 µM) with METTL16 silencing (sh-METTL16-2#, -3#). **B** The quantitative analysis of fluorescence intensity for granulosa cells. **C**, **D** The lipid peroxidation was initially detected and quantified using flow cytometry. **E** Ultrastructure images of the mitochondria in granulosa cells using TEM. **F** Mitochondria count number. Data are presented as mean ± SD (*n* = 3 independent biological replicates per group). *p < 0.05; **p < 0.01.

In addition to the accumulation of iron ions by METTL16 on granular cells, the following experiments continued to explore the effect of METTL16 on ROS. DCFH-DA results indicated that METTL16 silencing reduced the intracellular ROS (Figs. 4A, B). As ferroptosis was driven by the accumulation of lipid peroxidation accumulation, lipid peroxidation was initially detected and quantified using flow cytometry. As demonstrated in Figs. 4C, D, the lipid peroxidation was markedly decreased in METTL16 silencing. TEM was performed for cellular morphology analysis. TEM images indicated that METTL16 silencing improve cell mitochondria, membrane density and cristae (Figs. 4E, F). Overall, the data indicated that METTL16 silencing repressed the lipid peroxidation and alleviated the mitochondria injury.

### METTL16 targeted NRF2 to negatively regulated its RNA stability

**Fig. 5:**
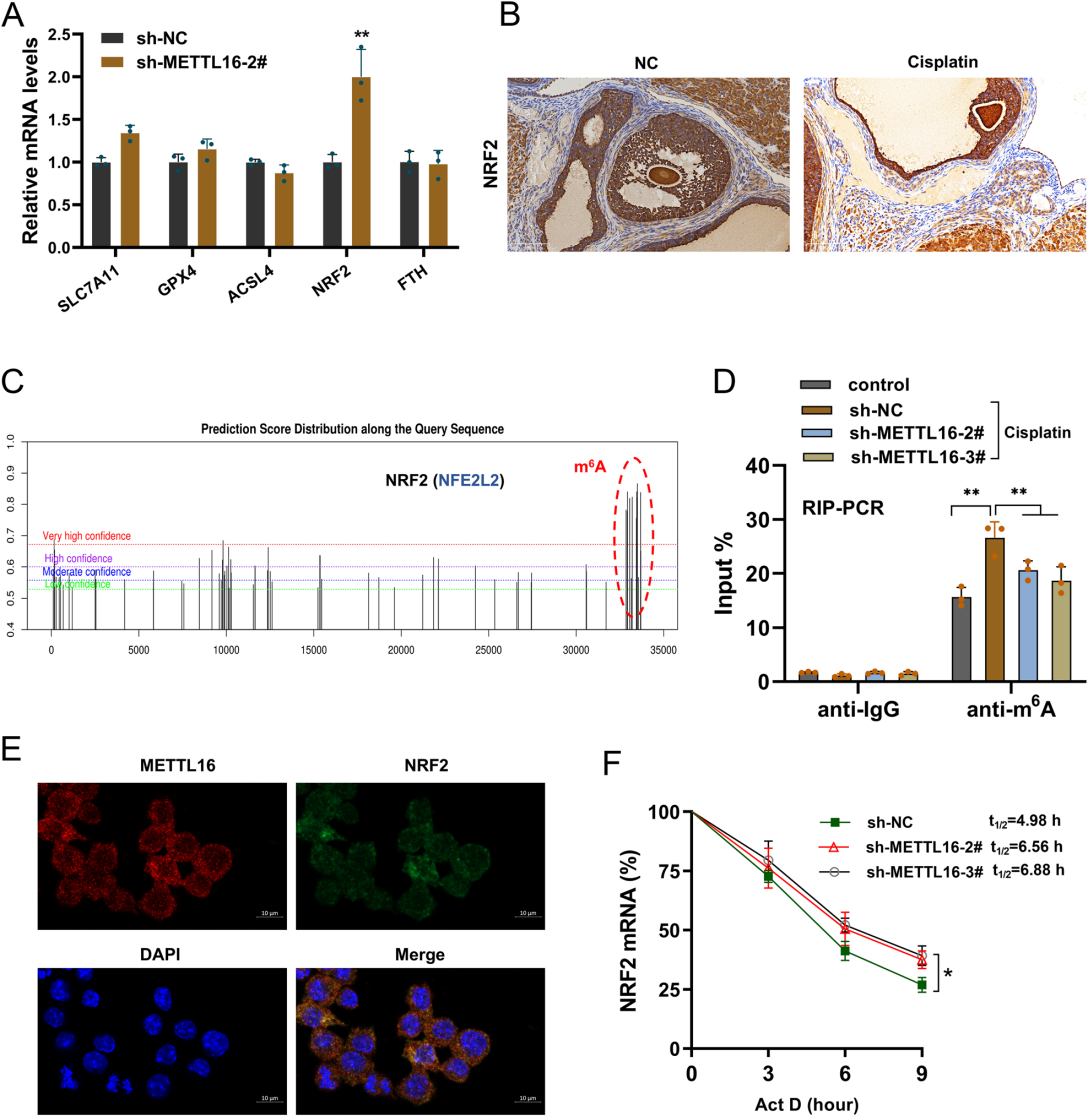
METTL16 targeted NRF2 to negatively regulated its RNA stability. **A** RT-PCR assays were performed to test the level of several genes associated with ferroptosis in granulosa cells with METTL16 silencing (sh-METTL16-2#). **B** The NRF2 was tested using immunohistochemical staining (IHC) in cisplatin-stimulated animal model ovarian tissue. **C** The online tools of m^6^A forecast (http://www.cuilab.cn/m6asiteapp/old) revealed the m^6^A modified sites on the 3’-UTR of NRF2 gene. **D** The immunoprecipitation assay by anti-m^6^A antibody revealed the m^6^A modified level of NRF2 mRNA in granulosa cells with METTL16 silencing (sh-METTL16-2#, sh-METTL16-3#). **E** The subcellular location of METTL16 and NRF2 in the granulosa cells by RNA fluorescence in situ hybridization. **F** RNA stability analysis revealed the NRF2 mRNA stability in granulosa cells with Act D treatment. Data are presented as mean ± SD (*n* = 3 independent biological replicates per group). *p < 0.05; **p < 0.01.

Given that METTL16 regulated the iron accumulation, lipid peroxidation and alleviated the mitochondria injury, the following assays were performed to investigate which pathway METTL16 specifically regulates ferroptosis. Several genes associated with ferroptosis were tested after METTL16 knockdown. The results showed that NRF2 had significant difference among these genes (Fig. 5A). The result prompted that NRF2 might be a downstream molecule of METTL16. In cisplatin-stimulated animal model ovarian tissue, the NRF2 was remarkably decreased (Fig. 5B). In the online tools of m^6^A forecast, there were numerous m^6^A modified sites on the 3’-UTR of NRF2 gene (Fig. 5C). For the m^6^A level of NRF2, the immunoprecipitation assay by anti-m^6^A antibody revealed that METTL16 silencing reduced the m^6^A modified level of NRF2 mRNA (Fig. 5D). The subcellular location of METTL16 and NRF2 revealed that them showed similar location in the granulosa cells (Fig. 5E). RNA stability analysis revealed that METTL16 silencing up-regulated the NRF2 mRNA stability in granulosa cells (Fig. 5F). In summary, the data revealed that METTL16 targeted NRF2 to negatively regulated its RNA stability.

### YTHDF2 synergized the degradation of NRF2 mRNA stability

**Fig. 6:**
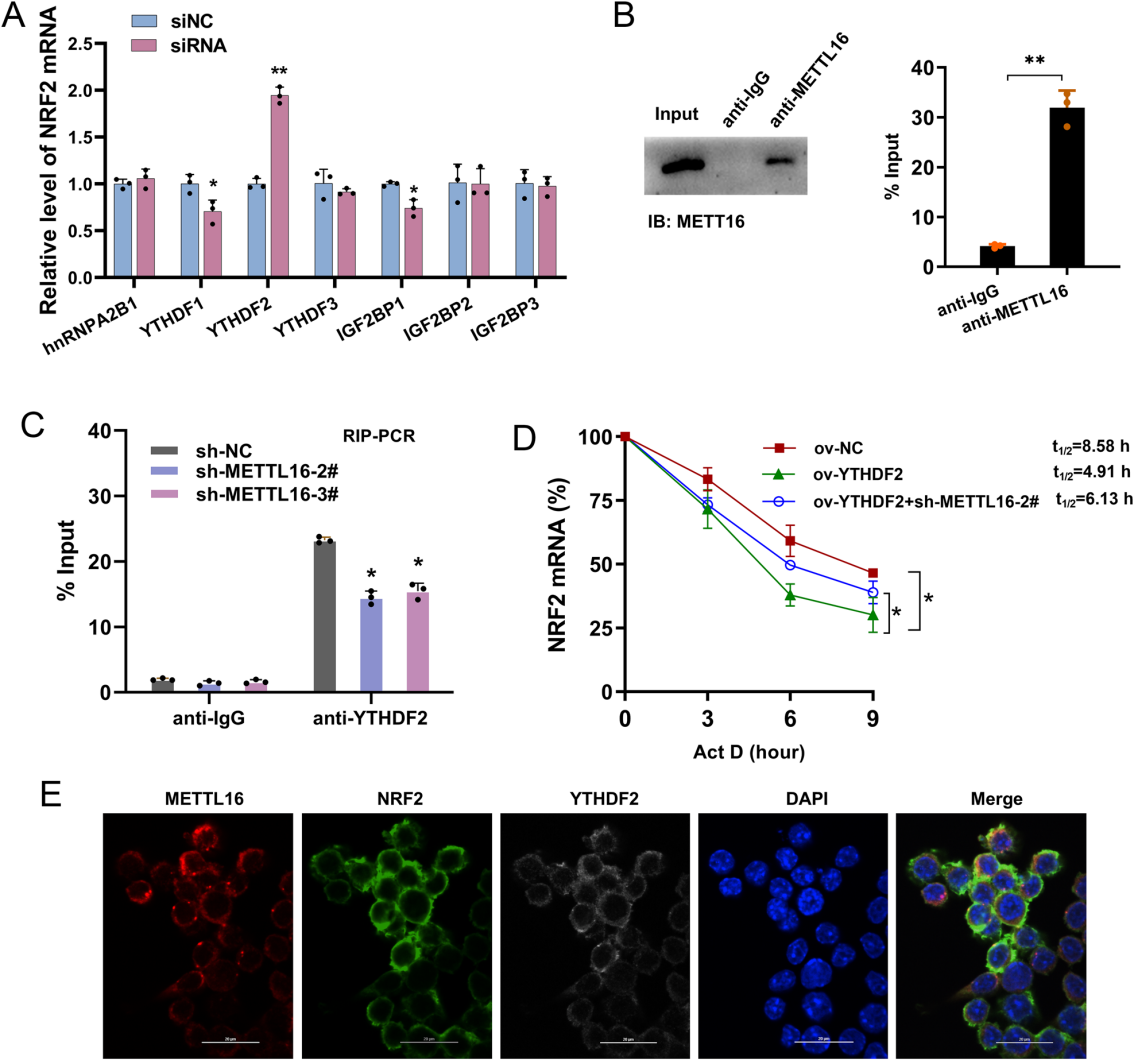
YTHDF2 synergized the degradation of NRF2 mRNA stability. **A** Numerous m^6^A readers were tested by the RT-PCR in granulosa cells that separately silenced by siRNA. **B** RIP-PCR analysis detected the interaction between METTL16 and NRF2 mRNA in granulosa cells. Enrichment of the precipitated RNA was measured by quantitative RT-PCR. Western blot of METTL16 showed high amount of input. Nonspecific IgG was acted as control. **C** The RIP-PCR analysis showed the interaction within YTHDF2 and NRF2 mRNA in granulosa cells with METTL16 silencing. **D** RNA stability analysis revealed the NRF2 mRNA stability in granulosa cells with Act D treatment. **E** The subcellular location of METTL16, NRF2 and YTHDF2 in the granulosa cells by RNA fluorescence in situ hybridization. Data are presented as mean ± SD (*n* = 3 independent biological replicates per group). *p < 0.05; **p < 0.01.

At present, there is a broad consensus that mRNA stability is mediated by m^6^A reader, so this study will try to find out which specific reader mediates NRF2 stability in the next study. Among the numerous m^6^A readers, the study selected the most representative reader proteins. Then, the m^6^A readers were separately silenced by siRNA. The results showed that only after YTHDF2 silencing, NRF2 had significant changes (Fig. 6A). RIP-PCR analysis showed that METTL16 interacted with NRF2 mRNA in granulosa cells (Fig. 6B). Then, the RIP-PCR analysis also showed that METTL16 silencing reduced the interaction within YTHDF2 and NRF2 mRNA in granulosa cells (Fig. 6C). RNA stability analysis revealed that YTHDF2 overexpression reduced the NRF2 mRNA stability, and METTL16 silencing co-transfection up-regulated the NRF2 mRNA stability (Fig. 6D). The subcellular location of METTL16, YTHDF2 and NRF2 revealed that them showed similar location in the granulosa cells (Fig. 6E). Therefore, the data revealed that YTHDF2 synergized the degradation of NRF2 mRNA stability.

### METTL16/YTHDF2/NRF2 axis regulated the ferroptosis of cisplatin-stimulated granulosa cells

**Fig. 7:**
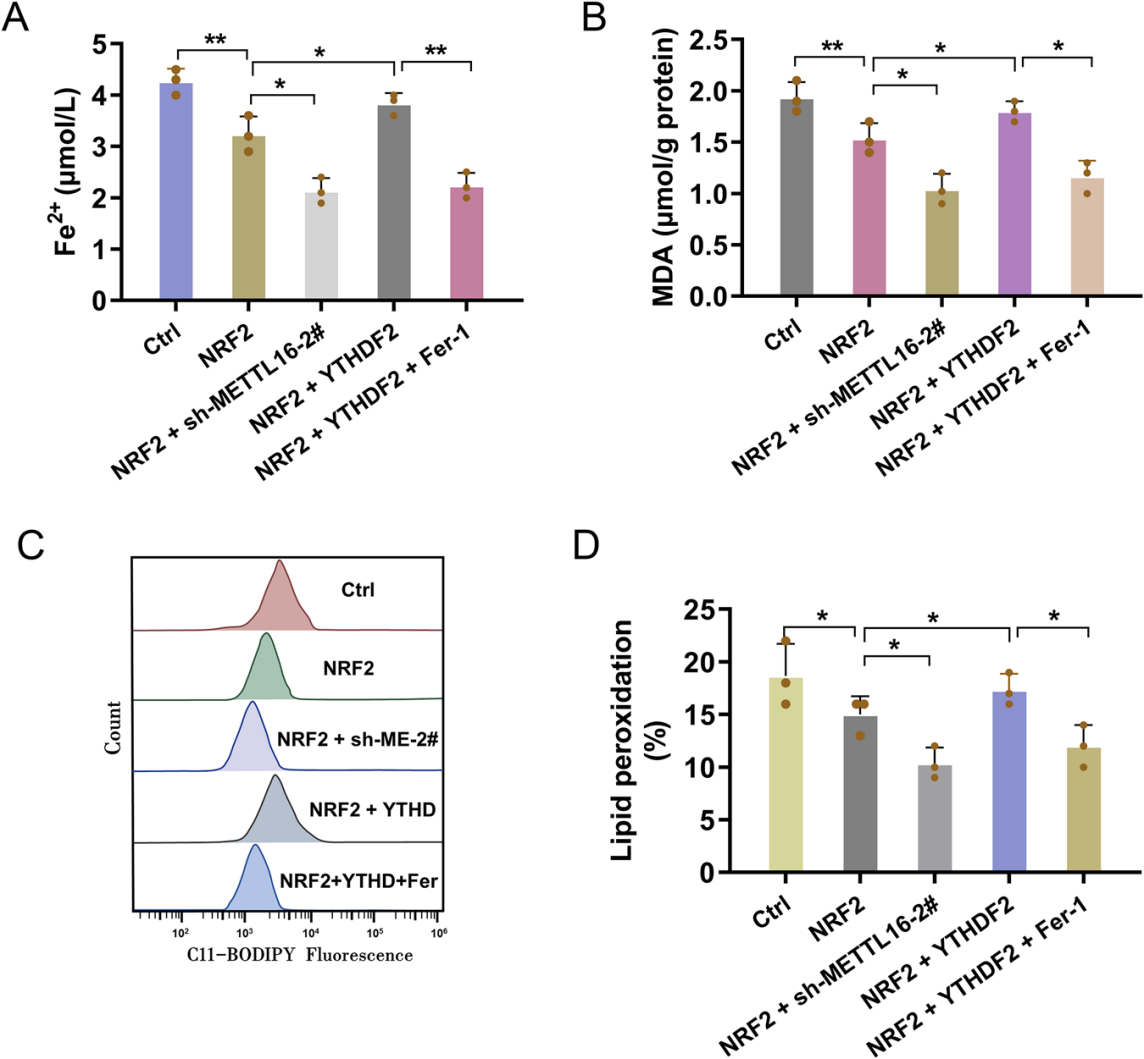
METTL16/YTHDF2/NRF2 axis regulated the ferroptosis of cisplatin-stimulated granulosa cells. **A** Fe^2+^ concentration was tested by the iron analysis kit. The granulosa cells were transfected with NRF2 overexpression (NRF2), METTL16 silencing (sh-METTL16-2#), YTHDF2 overexpression (YTHDF2), and selective ferroptosis inhibitor Ferrostatin 1 (Fer-1). **B** The MDA level was tested by the MDA kit. (**C**, **D**) The lipid peroxidation was initially detected and quantified using flow cytometry. Data are presented as mean ± SD (*n* = 3 independent biological replicates per group). *p < 0.05; **p < 0.01.

Next, the rescue assays were performed to test the role of METTL16/YTHDF2/NRF2 axis on the ferroptosis of cisplatin-stimulated granulosa cells. To assess the characteristics of ferroptosis, the results showed that NRF2 overexpression repressed the iron deposition (Fig. 7A), MDA (Fig. 7B), and lipid peroxidation (Figs. 7C, D). Moreover, METTL16 silencing aggravated the inhibitory effect of NRF2 on ferroptosis. Besides, YTHDF2 overexpression promoted the characteristics of ferroptosis. Ferrostatin 1 (Fer-1) is a potent and selective ferroptosis inhibitor. Fer-1 is a synthetic antioxidant that prevents damage to membrane lipids through a reductive mechanism, thereby inhibiting ferroptosis. Subsequently, Fer-1 could reduce the effect of co-transfection of NRF2 and YTHDF2 overexpression. In summary, METTL16/YTHDF2/NRF2 axis regulated the ferroptosis of cisplatin-stimulated granulosa cells.

## Discussion

**Fig. 8:**
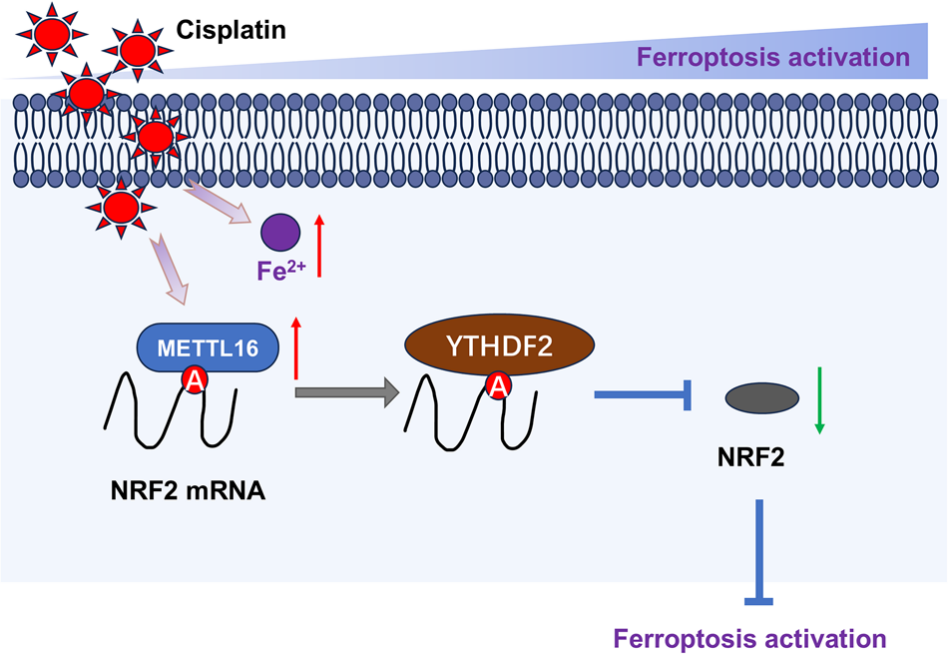
METTL16 accelerates the cisplatin-stimulated granulosa cell ferroptosis by YTHDF2/NRF2 axis in premature ovarian failure.

Premature ovarian failure (POF) is the most common reproductive endocrine disease that leads to low fertility or infertility in female at childbearing age [13]. Emerging studies have confirmed that follicle atresia or rapid depletion caused by abnormal granular cell status is an important pathological feature of POF [14–16]. In clinical treatment, the damage of female ovaries caused by drug toxicity caused by chemotherapy drugs, such as cisplatin, has been paid more and more attention. Therefore, this study explored the effects of epigenetic m^6^A RNA methylation on ovarian and granule cells (Fig. 8).

METTL16 is a recently identified m6A methyltransferase, composing of 562 amino acids [17–19]. The present study shows that the structure of METTL16 consists of four parts: RNA binding domain, methyltransferase domain, vertebrate conserved domain and potential nuclear localization sequence [20, 21]. METTL16 shows abnormal expression in series of diseases, and plays a different degree of promoting or inhibiting cancer in different pathological processes. For example, in acute myeloid leukemia, depletion of METTL16 dramatically inhibits the AML initiation and attenuates leukemia stem cells self-renewal [22]. Therefore, the role of METTL16 in pathophysiological process is attractive.

Presently, this work revealed that the level of METTL16 significantly elevated in the cisplatin-induced ovarian tissue. Besides, METTL16 was also up-regulated in cisplatin-stimulated granulosa cells. Functionally, METTL16 silencing repressed the iron accumulation and lipid peroxidation, and alleviated the mitochondria injury. These data illustrated that METTL16 functioned as a risk factor that accelerates the granule cells injury and ferroptosis.

During cisplatin-induced ovarian injury and ferroptosis of granulosa cells, there were significant changes for several genes or proteins. The m^6^A-dependent manner could significantly regulate the pathophysiological process [23]. Here, given that METTL16 might trigger the ferroptosis-related phenotypes, the following assays were performed to investigate the in-depth mechanism and target factors. Mechanistically, the data revealed that METTL16 targeted NRF2 mRNA to negatively regulated its RNA stability. Besides, m^6^A reader YTHDF2 synergized the degradation of NRF2 mRNA stability. NRF2 (nuclear factor erythroid-2 related factor 2), which is a key regulator of intracellular oxidation balance, has a wide range of antioxidant effects. Here, the results indicated that NRF2 impaired the ferroptosis in cisplatin-stimulated granulosa cells. METTL16/YTHDF2 axis repressed the level of NRF2, thereby accelerating the ferroptosis of granulosa cells. Therefore, m^6^A writer METTL16 might emerge as a new target for POF research and treatment.

It is noteworthy that METTL16 and NRF2 were co-localized not only in granulosa cells but also in oocytes, suggesting a potential broader role for the METTL16/YTHDF2/NRF2 axis within the follicle. While this study focused on granulosa cells due to their established vulnerability to cisplatin and central role in folliculogenesis, their expression in oocytes raises intriguing questions. Future studies should determine whether METTL16-mediated m^6^A modification directly regulates NRF2 in oocytes, thereby influencing oocyte quality or chemoresistance. Employing oocyte-specific models will be essential to dissect the cell-autonomous role of this axis and to develop comprehensive therapeutic strategies for POF.

While this study primarily elucidates the causal role of the METTL16/YTHDF2/NRF2 axis in regulating ferroptosis of granulosa cells at the cellular level, it is important to acknowledge its limitations. Although data from animal models (e.g., IHC) show a strong correlation between key molecules of this axis and POF phenotypes, we were unable to directly validate whether targeting METTL16 could alleviate cisplatin-induced ovarian failure in *vivo*, due to constraints in funding, time, and experimental models. The lack of in vivo functional evidence through genetic knockout or inhibition remains a gap to be addressed in future studies. We intend to make this a focus of subsequent work, employing models such as conditional knockout mice to further evaluate the interventional potential of this axis in vivo.

In summary, the work revealed the role of METTL16 on cisplatin-stimulated granulosa cells in POF. METTL16/YTHDF2/NRF2 axis regulated the ferroptosis of cisplatin-stimulated granulosa cells in POF. Therefore, METTL16 might serve as a promising immunotherapeutic strategy for POF.

## Materials and methods

### Cells and culture

Human ovarian granulosa KGN cells (Procell, cat. CL-0603). KGN cells were cultured in 6-well culture plates by high-sugar DMEM added with1% penicillin/streptomycin, 10% FBS at 5% CO_2_ and 37 °C. For POF cellular model, cells were treated with the concentration of cisplatin (CDDP, 5 µM, 10 µM, 20 µM, Sigma-Aldrich).

### Animals

Cisplatin-induced premature ovarian failure (CDDP-POF) model was constructed according to the previous study [24]. Female mice (6–8 weeks, *n* = 10) were treated by intraperitoneal injection of cisplatin (2 mg/kg, 7-day). The controls (*n* = 10) were treated by an equal volume of saline solution. After 7 days, the mice were euthanized and ovarian tissue was collected. All animal experiments were approved by the Ethical Committee of the Second Xiangya Hospital of Central South University (Approval No. 2021428) and were performed in accordance with the relevant guidelines and regulations for the care and use of laboratory animals.

### ELISA and ethynyl deoxyuridine (EdU) incorporation assays

The concentrations of estradiol (E2), anti-Müllerian hormone (AMH) and follicle-stimulating hormone (FSH) were measured using enzyme-linked immunosorbent assay following the manufacturer’s instructions. The serum levels were determined using a standard curve. In each group, the absorbance was recorded at 450 nm. For the EdU analysis, the EdU incorporation assay was carried out with an EdU kit (Roche, Mannheim, Germany) in accordance with the manufacturer’s instruction.

### Transfection

The three highest-scoring shRNA sequences targeting human METTL16 were designed and synthesized (pHBLV-U6-MCS-CMV-ZsGreen, Hanbio, Shanghai, China), including sh-METTL16-1#, sh-METTL16-2#, sh-METTL16-3#. Empty vectors were used as a negative control. For YTHDF2 overexpression transfection, granulosa cells were transfected with Lipofectamine 2000 reagent (Invitrogen, Carlsbad, CA, USA) according to the manufacturer’s instruction.

### Western blot

Cells were washed on ice-cold by PBS in RIPA buffer (Thermo Fisher Scientific) containing 1 mM PMSF (Thermo Fisher Scientific). Whole-cell extracts were collected and protein concentration was determined by BCA. The lysates were subjected to sodium dodecyl sulfate-polyacrylamide gel electrophoresis (10%, SDS-PAGE) and transferred to PVDF membranes (Millipore, MA, USA). The membrane was then blocked with PBS with non-fat dried milk (5%). PVDF membranes were incubated with primary antibodies overnight (anti-METTL16, Cat No. 19924-1-AP, 1:1000). After incubation by HRP-conjugated secondary antibody, blots were visualized with ECL detection system (Millipore).

### Fe^2+^, GSH and MDA detection

Fe^2+^ level was detected following the instructions of the iron assay kit (Sigma). Malondialdehyde (MDA) was assayed according to the instructions of MDA Assay Kit (Beyotime). GSH level was assayed according to the instructions of GSH Assay Kit (Beyotime).

### FerroOrange

Intracellular iron level was determined using FerroOrange assay. After treatment, cells were stained with FerroOrange (1 μmol/L) at 37 °C for 30 min in the dark. Confocal laser scanning microscope (ZEISS) was performed to acquire images. Fluorescence intensity was analyzed using ImageJ software.

### ROS detection of DCFH-DA assay

Intracellular ROS level was assessed using DCFH-DA (ApexBio, USA). Cells were treated and then stained with DCFH-DA (50 μmol/L) for 30 min in the dark. Images were captured using a confocal laser scanning microscope (ZEISS). Fluorescence intensity was measured with ImageJ and normalized to the average intensity related to control group.

### Lipid peroxidation measurement

The lipid peroxidation level was detected by the oxidation-sensitive probe C11-BODIPY581/591 (Thermo, D3861, 5 μM) for 20 min. Then, the cells were also stained by DAPI. Intracellular fluorescence intensity was tetsted using flow cytometer (D Biosciences, USA).

### Transmission electron microscopy

The cells were treated with transfection, and then the cells were collected and centrifuged. Subsequently, cells were fixed by electron microscope fixation solution. The sample was embedded and sectioned and stained by uranyl acetate and lead citrate. The images were observed and photographed with transmission electron microscope (Hitachi HT-7700).

### RNA-fluorescence in situ hybridization (FISH)

Cy3-labeled METTL16, FAM-labeled NRF2 and DAPI-labeled probes were obtained from Genepharma (Shanghai, China). FISH was performed using fluorescent in situ hybridization kit according to the manufacturer’s protocol.

### RNA stability

Granulosa cells were seeded in 6-well plates (1 × 10^5^ cells per well). 24 h later, cells were exposed to Actinomycin D (2 μg/ml, Act D, Sigma) and then collected at indicated time point. The half life time was analyzed using qRT-PCR and normalized to control.

### RNA immunoprecipitation (RIP) assay

For the m^6^A RNA binding, the RNA of granulosa cells with transfection were isolated and treated with deoxyribonuclease I. RNAs were fragmented by sonication on ice water. Immunoprecipitation was performed using anti-YTHDF2 (Cat No. 24744-1-AP, Proteintech, 1:500), anti-m^6^A antibody (Cat No. 68055-1-Ig, Proteintech, 1:1000). Magnetic Dynabeads was bound to the RIP Immunoprecipitation Buffer. After incubation, fragmented RNAs were treated with proteinase K. RNAs was extracted and subjected to qRT-PCR using primers normalized to input.

### RNA sequencing

Total RNA was extracted from ovarian tissues of the cisplatin-treated group and control group using TRIzol reagent (Invitrogen). The sequencing was performed by Illumina NovaSeq™ 6000. The differentiated transcriptional level was analyzed by R package edgeR based on log2 (fold change)≥1 and *p* < 0.05.

### Statistical analysis

Statistical analyses and visualizations were conducted using GraphPad Prism version 9.0 software (GraphPad Software). The two-tailed *t*-test was performed for two groups. The one-way analysis of variance (ANOVA)-Dunnett’s test was performed for multiple groups. Quantitative data was presented using the Mean ± SD. *P*-value < 0.05 was considered as statistically significant.

## Supplementary information

BLOT Supplemental Material

## Data Availability

The datasets generated during and/or analysed during the current study are available from the corresponding author on reasonable request.
