# Supplementary figures and images for: Silencing of METTL16 protects granulosa cells from the cisplatin-induced ferroptosis in premature ovarian failure
Source: Cell Death Discov. 2026 Apr 19;12:256. doi: 10.1038/s41420-026-03081-3 (PMC13219508; doi:10.1038/s41420-026-03081-3)

## Supplemental Material

Fig 2 blot-1 beta-actin

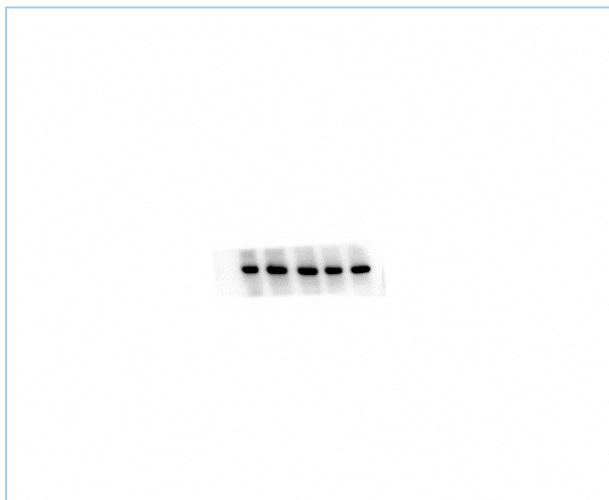

Fig 2 blot-2 METTL16

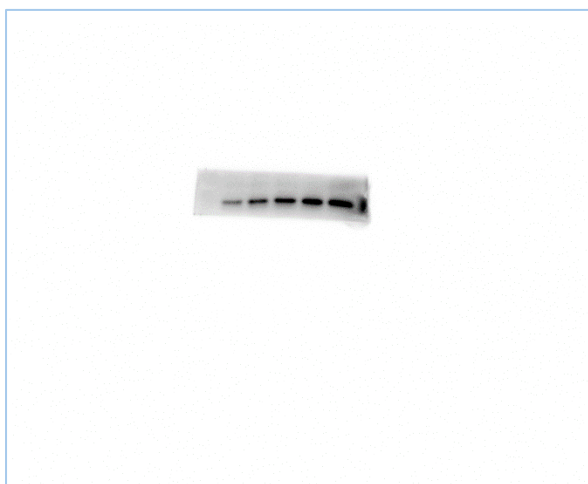

**Fig 3 blot-1    beta-actin**

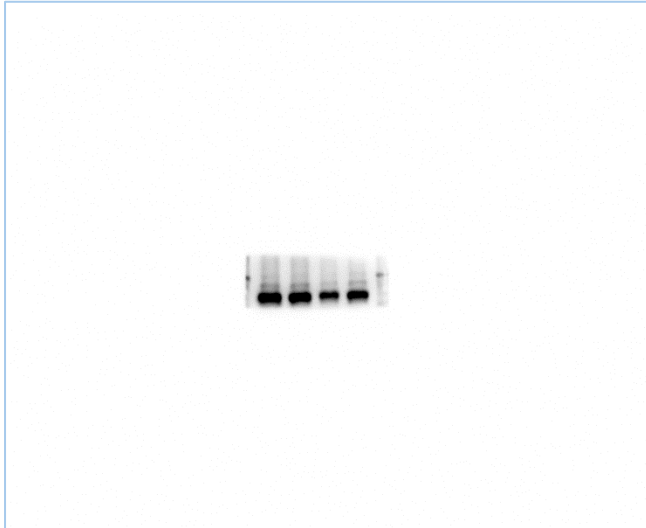

**Fig 3 blot-2    METTL16**

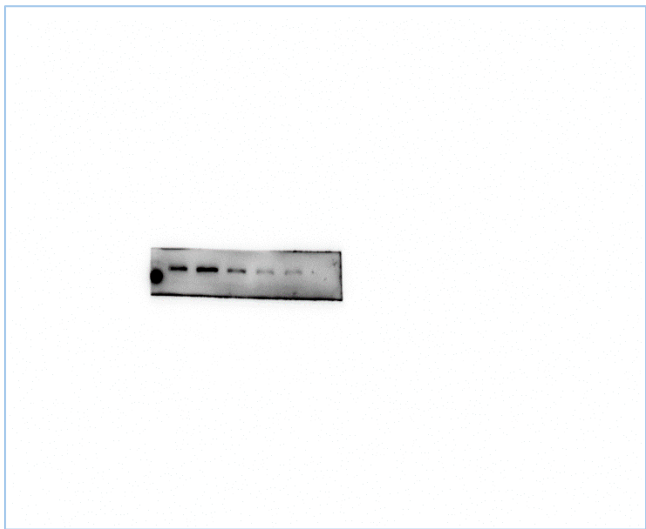

Supplement: Supplementary file 1 — BLOT Supplemental Material [file 41420_2026_3081_MOESM1_ESM.pdf]
